# Management of Complex Three-Wall Orbital Fractures with Digital Planning and Patient-Specific Two-Piece Implants

**DOI:** 10.3390/medicina62061043

**Published:** 2026-05-28

**Authors:** Lisa Catarzi, Giulio Cirignaco, Massimiliano Gilli, Roberto Lo Giudice, Maurizio Gladi, Carlos M. Chiesa-Estomba, Luigi Angelo Vaira, Giuseppe Consorti

**Affiliations:** 1 Maxillo-Facial Surgery Unit, Department of Medicine, University of Siena, Viale Bracci, 53100 Siena, Italy; g.cirignaco@student.unisi.it; 2 Department of Maxillo-Facial Surgery, Santa Maria Della Misericordia Hospital, 06132 Perugia, Italy; massimilianogilli0@gmail.com; 3 Department of Human Pathology of Adults and Developmental Age, Messina University, 98100 Messina, Italy; roberto.logiudice@unime.it; 4 Neurosurgical Department, Marche Polytechnic University, Azienda Ospedaliero Universitaria delle Marche, 60126 Ancona, Italy; maurizio.gladi@ospedaliriuniti.marche.it; 5 Department of Otorhinolaryngology-Head and Neck Surgery, Hospital Universitario Donostia, 20003 San Sebastian, Spain; chiesaestomba86@gmail.com; 6 Maxillofacial Surgery Operative Unit, Department of Medicine, Surgery and Pharmacy, University of Sassari, 07100 Sassari, Italy; lavaira@uniss.it; 7 Division of Maxillofacial Surgery, Department of Neurological Sciences, Marche University Hospitals-Umberto I, 60126 Ancona, Italy; giuseppe.consorti@ospedaliriuniti.marche.it

**Keywords:** orbital reconstruction, orbital fracture, patient-specific implant, virtual surgical planning

## Abstract

*Background and Objectives*: The surgical management of three-wall orbital fractures remains a significant challenge due to complex anatomy, limited exposure, and the absence of clear landmarks. These extensive reconstructions are rare and traditionally burdened by high complication rates and inconsistent outcomes. This study presents a standardized surgical protocol for complex three-wall orbital reconstruction, highlighting the role of digital planning and a novel two-piece interlocking patient-specific implant (PSI). *Materials and Methods*: Between 2018 and 2024, 17 patients with unilateral three-wall orbital fractures underwent reconstruction using digitally planned, patient-specific two-piece titanium implants designed to restore the orbital floor, medial, and lateral walls. Implant positioning was assessed through qualitative evaluation of postoperative CT scans and quantitative comparison between planned and actual implant positions, as well as orbital volume analysis between reconstructed and unaffected orbits. Clinical outcomes were evaluated pre- and postoperatively. *Results*: Reconstruction was classified as ideal in 16 cases (94.1%) and satisfactory in one case (5.9%). Quantitative analysis demonstrated a high level of concordance between the planned and postoperative implant positions, with a mean deviation of 0.982 ± 0.107 mm (95% CI: 0.927–1.037 mm). All implants were positioned within 1.5 mm of the planned location. Postoperative orbital volumes closely approximated those of the contralateral side, with a mean volume difference of 1.371 ± 0.176 cm^3^ (95% CI: 1.280–1.461 cm^3^). Diplopia resolved in all patients, and enophthalmos was fully corrected in 15 cases (88.2%). No major complications or revision surgeries were observed. *Conclusions*: The proposed two-piece interlocking PSI enabled precise and reproducible reconstruction of complex three-wall orbital fractures. This approach demonstrates that even technically demanding orbital reconstructions can be performed with greater reliability, leading to favorable functional and aesthetic outcomes.

## 1. Introduction

The surgical management of extensive orbital fractures remains one of the most challenging aspects of maxillofacial trauma surgery due to the complex three-dimensional anatomy of the orbit, limited surgical exposure, and the frequent absence of reliable anatomical landmarks. Fractures involving three orbital walls represent a distinct and severe subset of orbital injuries, often associated with marked disruption of the bony framework, significant orbital volume increase, and a high risk of persistent functional and aesthetic sequelae, including diplopia and enophthalmos [1].

Although isolated orbital floor or inferomedial fractures are relatively common and can be managed successfully using preformed titanium meshes [2], multi-wall fractures are considerably less frequent and are associated with substantially higher rates of implant malposition and suboptimal reconstruction [3]. Even minimal inaccuracies in implant positioning or contour restoration may translate into clinically relevant changes in orbital volume, which are poorly tolerated in three-wall defects and may compromise long-term outcomes.

Over the past decade, progressive refinements in orbital exposure techniques, implant materials, and surgical expertise have improved the overall quality of orbital reconstruction [4,5]. In parallel, digital preoperative planning and computer-assisted design have emerged as valuable tools for addressing complex orbital defects [6].

Patient-specific implants (PSI), generated through virtual surgical planning and three-dimensional modeling, enable precise restoration of patient anatomy and have demonstrated superior accuracy compared with non-patient-specific implants in selected clinical scenarios [7,8]. A recent systematic review and meta-analysis by Kotecha et al. [9] reported a trend toward improved postoperative orbital volume restoration, reduced enophthalmos, and shorter operative times with the use of patient-specific implants (PSIs) compared with conventional techniques. However, these differences did not reach statistical significance, suggesting that current evidence does not yet confirm the superiority of PSIs. Consequently, the selection of the reconstructive material should remain at the surgeon’s discretion, guided by clinical context and intraoperative findings [9,10].

When defects extend beyond the orbital floor and medial wall to include the lateral wall, conventional preformed implants often prove inadequate, and even single-piece PSI may be difficult to insert and position accurately through limited surgical corridors [11]. In three-wall fractures, the need to reconstruct multiple discontinuous surfaces simultaneously, combined with restricted access and increased manipulation of orbital soft tissues, further increases surgical complexity and the risk of iatrogenic injury [12,13,14].

Despite these challenges, the current literature provides limited guidance on standardized surgical strategies or implant designs specifically tailored to post-traumatic three-wall orbital reconstruction, and reported complication rates and inconsistent reconstructive success remain high [13,14,15].

In this context, we present a novel interlocking two-piece patient-specific implant and a standardized digital workflow for the reconstruction of complex three-wall orbital fractures. This modular design aims to facilitate controlled implant insertion, enhance intraoperative spatial orientation, and improve the reproducibility and accuracy of orbital reconstruction in cases traditionally considered highly challenging.

## 2. Materials and Methods

### 2.1. Study Design and Patient Selection

Between 2018 and 2024, clinical data from patients with complex three-wall orbital fractures who underwent reconstruction of the concomitant orbital floor, medial, and lateral walls were retrospectively collected and analyzed. All procedures were performed at tertiary referral centers by the same senior surgeon (G.C.), with more than 10 years of experience in orbital reconstruction. Inclusion criteria were: (i) age ≥ 18 years at the time of surgery; (ii) unilateral post-traumatic fracture involving the orbital floor, medial wall, and lateral wall; (iii) availability of high-resolution preoperative and postoperative CT scans; and (iv) a minimum clinical follow-up of 12 months. Exclusion criteria included previous orbital trauma or orbital disease, bilateral orbital fractures, need for emergency decompression (e.g., retrobulbar hematoma), and incomplete imaging or follow-up data.

### 2.2. Imaging Protocol

All patients underwent CT scanning with a slice thickness of 0.6 mm pre- and postoperatively. DICOM datasets were used for virtual surgical planning, implant design, and postoperative assessment. Postoperative CT scans were routinely obtained within 72 h after surgery to verify implant positioning and orbital reconstruction accuracy.

### 2.3. Digital Surgical Planning and Implant Design

A fully digital workflow was adopted for all cases. Virtual surgical planning was performed using dedicated software (Brainlab Elements Contouring, version 4.0, Brainlab, Feldkirchen, Germany). The unaffected orbit was segmented and mirrored onto the injured side to generate a three-dimensional reference model representing the patient’s native orbital anatomy.

Based on this virtual reconstruction, a two-piece patient-specific implant (Sintac s.r.l. Biomedical Engineering, Trento, Italy) was designed by a maxillofacial surgeon in collaboration with an engineer. One component (inferomedial implant) was designed to reconstruct the orbital floor and medial wall contours as a single unit, while the second component (lateral implant) reproduced the lateral orbital wall. Each component was modeled to rest on intact bony margins to ensure passive seating and mechanical stability. The two plates were intentionally designed to be inserted separately to reduce incision length.

The inferomedial implant is designed first. It extended anteroposteriorly from the infraorbital margin to the posterior edge of the orbital process of the palatine bone. Medially, the superior medial border of the plate was planned to abut or slightly extend beyond the fracture margins of the medial wall.

**Figure 1:**
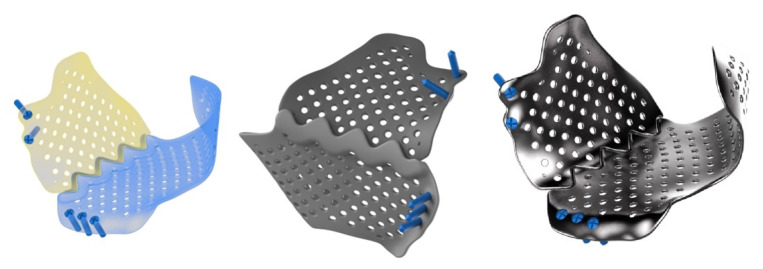
Three-dimensional computer-aided design of the two-piece implant for reconstruction of three orbital walls. The two components (yellow and blue) interlock with each other.

The interface between the two components was positioned at the inferior orbital fissure and featured a male–female interlocking mechanism with overlapping contact surfaces, allowing precise intraoperative co-localization, tactile feedback during assembly and mechanical stability (Figure 1).

The lateral implant was planned to extend slightly beyond the fracture margins of the lateral wall, thereby recreating the contour of the healthy contralateral orbit.

Both components incorporated preplanned fixation points: the inferomedial implant along the infraorbital rim and the lateral implant at the frontozygomatic process. Implant thickness and contours were tailored to replicate the mirrored anatomy while minimizing implant bulk. All implants were manufactured in titanium alloy using additive manufacturing techniques and were supplied as sterile, ready-to-use devices compliant with current European regulatory standards. No intraoperative bending or reshaping was required due to the patient-specific design.

### 2.4. Surgical Technique

All procedures were performed under general anesthesia. Orbital exposure was achieved through a transconjunctival preseptal approach combined with a medial transcaruncular incision and inferior cantholysis/canthotomy when required. This approach allowed adequate visualization of the orbital floor, medial wall, and lateral wall while avoiding transcutaneous incisions.

Dissection of the orbital floor and medial and lateral walls was performed to expose and identify all fracture margins. The inferomedial implant was inserted first using controlled rotational and sliding movements until passive and stable seating on healthy bone was achieved. All plate margins were carefully inspected. The plate was then fixed with one screw, ensuring that the pilot hole was perfectly centered.

Next, the lateral implant was positioned and interlocked with the inferomedial component through the predesigned interface. Final fixation was completed by securing both components to the orbital rim. No intraoperative implant adjustments were necessary.

### 2.5. Post-Operative Outcome Assessment

Postoperative implant positioning was independently evaluated by two senior surgeons using axial, coronal, and sagittal CT images. Implant placement was classified according to previously described criteria [16] as ideal (implant resting on sound bony margins with restoration of normal contour), satisfactory (restoration of normal contour without full contact on all bony margins), acceptable (contact with most healthy margins but incomplete contour restoration), or poor (contact with only one or two intact margins, implant edge displaced outside the orbital contour, and failure to restore anatomy).

For quantitative analysis, planned and postoperative implant positions were superimposed using Brainlab Elements Contouring version 4.0 (Brainlab, Feldkirchen, Germany), and mean linear deviations were calculated.

Orbital volume was assessed through semi-manual segmentation of both the reconstructed and contralateral orbits. Differences between sides were calculated to evaluate volumetric restoration. Deviations within 1.5 mm and limited volume discrepancies were considered clinically acceptable based on established orbital reconstruction standards [17]. Postoperative CT imaging was performed early (<72 h) to assess implant positioning accuracy and anatomical reconstruction before any confounding effects related to bone remodeling or soft tissue adaptation.

**Figure 2:**
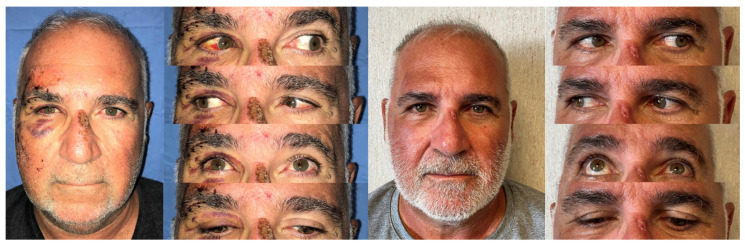
Preoperative (**left**) and postoperative (**right**) clinical photographs of a representative patient. Postoperative evaluation demonstrates restoration of globe projection and orbital contour symmetry, with stable eyelid position. Ocular motility was clinically full in the cardinal gaze positions, and no diplopia was detected.

Clinical outcomes were assessed pre- and postoperatively with a minimum of follow up of 12 months. All patients underwent standardized ophthalmologic evaluation pre- and postoperatively. Ocular motility was clinically assessed in primary gaze, upgaze, downgaze, and lateral gaze positions Figure 2. Diplopia was evaluated through patient-reported symptoms and confirmed by clinical examination of extraocular movements in the cardinal gaze positions and orthotic evaluation with Hess-Lancaster test.

Resolution of diplopia was defined as the absence of double vision in all visual fields. Enophthalmos was considered clinically relevant when exceeding 2 mm.

### 2.6. Statistical Analysis

Categorical variables were reported as frequencies and percentages, while continuous variables were expressed as mean values and standard deviations. Implant positioning accuracy and orbital volume differences were primarily analyzed using descriptive statistics, including mean values, standard deviations, and 95% confidence intervals.

Orbital volume comparison between reconstructed and unaffected orbits was performed using a paired two-tailed Student’s *t*-test after assessment of data normality with the Shapiro–Wilk test. Statistical analyses were conducted using SPSS software (version 20.0; SPSS Inc., Chicago, IL, USA).

## 3. Results

Seventeen patients fulfilled the inclusion criteria and were included in the analysis.

The mean age at presentation was 33.6 years (SD, 10.4 yr; range, 22–53 yr), 14 patients (82.4%) were male and 3 (17.6%) female. With respect to mechanism, the majority of the fractures were caused by road traffic accidents (*n* = 10, 58.8%), assault (*n* = 4, 23.5%), sporting injuries (*n* = 2, 11.8%), followed by job injuries (*n* = 1, 5.9%). Multiple walls (orbital floor, medial, and lateral walls) were involved in all case (Table 1).

**Table 1:** Demographic and Trauma Characteristics of the Study Population.

Variable	Value
**Mean age (years)**	33.6 ± 10.4 (range 22–53)
**Sex**	
Male	14 (82.4%)
Female	3 (17.6%)
**Mechanism of injury**	
Road traffic accident	10 (58.8%)
Assault	4 (23.5%)
Sport-related injury	2 (11.8%)
Occupational injury	1 (5.9%)
**Fracture Type**	
Orbital Floor + medial wall + lateral wall	17 (100%)

### 3.1. Radiological Accuracy

**Figure 3:**
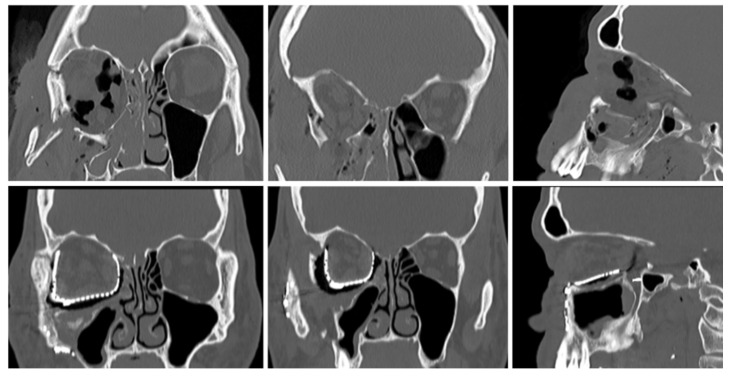
Pre and post-operative computed tomography scans showing reconstruction of the three-walled orbital fracture. In this case, the reconstruction result was assessed as “ideal”.

Postoperative CT scans confirmed accurate anatomical reconstruction in all cases. The reconstruction was considered ideal in 16 cases (94.1%) (Figure 3) and satisfactory in 1 case (5.9%).

Given the descriptive and feasibility-oriented nature of this study, implant positioning accuracy was interpreted primarily from a clinical perspective rather than through hypothesis-driven statistical testing. Deviations were therefore reported using descriptive metrics and clinically meaningful thresholds. Quantitative assessment of implant positioning demonstrated a high degree of concordance between the virtual surgical plan and the final postoperative outcome. The mean implant deviation was 0.982 mm (SD 0.107 mm), with a 95% confidence interval (CI) of 0.927–1.037 mm. All implants were positioned within 1.5 mm of the preoperative plan.

**Figure 4:**
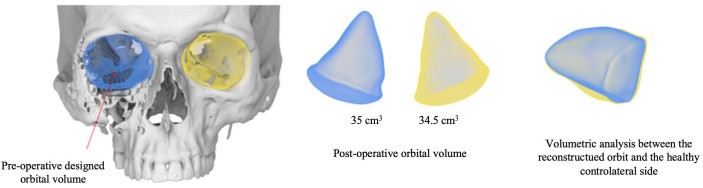
Orbital Volume Comparison; blue: reconstructed side, yellow: unaffected side.

Volumetric analysis revealed a close correspondence between the reconstructed orbit and the healthy contralateral side. The mean absolute postoperative volume difference was 1.371 cm^3^ (SD 0.176 cm^3^), with a 95% CI of 1.280–1.461 cm^3^ (Figure 4).

### 3.2. Clinical Outcomes

Clinical outcomes were assessed at a follow-up of 12 months, focusing on functional recovery and globe position stability. At presentation, all patients exhibited diplopia and clinically relevant enophthalmos (>2 mm). At 12-month follow-up, diplopia had resolved completely in all cases (0%, 0/17), both subjectively and on formal orthoptic examination. Enophthalmos was fully corrected in 15 patients (88.2%), while 2 patients (11.8%) demonstrated substantial improvement, achieving a clinically acceptable globe position despite minimal residual enophthalmos. No major postoperative complications were observed, and no revision surgery was required in this cohort. Details of outcome results are summarized in Table 2.

## 4. Discussion

Reconstruction of extensive three-wall orbital fractures represents one of the most technically demanding challenges in orbital trauma surgery. These injuries are characterized by severe disruption of the bony framework, loss of reliable anatomical landmarks, and restricted working corridors for implant placement, all of which contribute to a higher risk of implant malposition and unsatisfactory functional or aesthetic outcomes. Moreover, the need for precise anatomical restoration is amplified in this subset of fractures, which exhibit the highest rates of clinically significant enophthalmos, reaching up to 85.2% [1].

Despite the clinical relevance of these injuries, only a limited number of studies have specifically addressed the reconstruction of orbital defects extending beyond the medial wall and floor [2,4,13,18].

The present study introduces and evaluates a modular interlocking two-piece patient-specific implant (PSI) designed specifically for post-traumatic three-wall orbital reconstruction. By combining digital preoperative planning with a patient-tailored implant geometry, this approach aims to improve spatial control during implant placement and enhance the reproducibility of reconstruction in highly complex cases. The consistently favorable radiological and clinical outcomes observed in this series support the feasibility and reliability of this strategy.

Although preformed titanium meshes remain the standard of care for many orbital fractures, their limitations become evident in subtotal orbital defects involving multiple walls. In such scenarios, non-patient-specific implants may be difficult to adapt accurately, particularly when surgical exposure is limited, and may fail to restore the complex three-dimensional orbital contour [11].

Digital preoperative planning allows surgeons to perform a detailed analysis of fracture morphology, to simulate ideal orbital anatomy, and to generate an implant tailored to the patient’s native contours. While a single-piece PSI could theoretically reconstruct multi-wall defects, insertion and positioning of a large monobloc implant often require wide exposure and extensive manipulation of orbital soft tissues. In contrast, the two-piece modular configuration described in this study enables sequential and controlled insertion of smaller components through limited access. The inferomedial component restores the orbital floor and medial wall, while the lateral component reconstructs the lateral wall, with accurate co-localization ensured by the interlocking interface at the inferior orbital fissure. This design offers a more favorable balance between access, accuracy, and operative safety [11,19,20] and provides intraoperative tactile feedback minimizing the risk of cumulative positioning errors.

To date, two-piece PSI have primarily been described for extensive orbital floor defects [21,22,23] or inferomedial two-walled fractures [19,20,21] where separate components reconstruct the floor and medial wall.

To our knowledge, only two publications have reported multi-piece PSI reconstruction of subtotal orbital defects.

Sabelis et al. [14] described three- and four-walls PSI reconstruction in a non-traumatic oncologic case of spheno-orbital meningioma, using ridges and hook-and-bar connectors to align two orbital components. Although acceptable in four-wall oncologic reconstruction, these tolerance-allowing connectors may introduce unnecessary inaccuracy in trauma-related three-wall defects requiring maximal precision. 

Pinto et al. reported PSI reconstruction of a post-traumatic four-wall defect due to gunshot injury, in which the frontozygomatic pillar was missing, representing extensive osseous loss secondary to the gunshot injury [18].

Their three-piece PSI reconstructed all four walls and additionally replaced the nasomaxillary buttress, frontozygomatic process, and inferior orbital rim, functioning as a sort of true prosthetic substitute. As a result, very extensive surgical exposures were required, involving an extended infraorbital approach for the medial wall/floor and lateral wall component, and a bicoronal approach for the roof component. Twenty-three screws were necessary to stabilize the implants, and no specific design features were provided to assist intraoperative co-localization of the components.

Our implant design and surgical strategy differ from previously reported approaches. We developed a modular system consisting of only two components: a single inferomedial plate reconstructing both the orbital floor and medial wall, and a second plate restoring the lateral orbital wall. This design builds on our prior experience with single-piece inferomedial patient-specific implants, which demonstrated that combined reconstruction of the floor and medial wall can be reliably achieved through limited surgical access, with excellent outcomes and no additional morbidity [5]. Leveraging this evidence, the inferomedial component was intentionally designed to restore these two walls, while a dedicated second component was developed for lateral wall reconstruction.

A key advantage of this configuration is the independent insertion of the inferomedial and lateral components, which minimizes the need for wide surgical exposure. Unlike tolerance-allowing connectors or complex multi-component constructs described in oncologic or extreme traumatic reconstructions, the overlapping interlocking mechanism adopted in this system ensures stable and precise alignment without increasing implant bulk or requiring extended surgical approaches. This modular strategy enables controlled, sequential placement of components that would be difficult—or impractical—to insert as a single unit, thereby reducing repeated insertion attempts and limiting manipulation of orbital contents. As a result, the entire procedure can be performed through transconjunctival and transcaruncular access, avoiding external scars and reducing surgical morbidity.

A critical intraoperative step is confirming that the first component seats passively on stable bony margins without any “bounce,” as the positioning of the second component depends on the accuracy of the first [4].

Compared with previous designs described by Sabelis and Pinto [14,18], our interlocking mechanism incorporates an overlapping interface along the entire connection length, ensuring stable and precise alignment while increasing the surface area of contact. As in other PSI systems, both components are secured to the orbital rim with 2–3 screws each.

Overall, the two-piece modular PSI used in this study addresses several limitations of traditional techniques by enabling controlled insertion, minimizing soft-tissue manipulation, reducing the number of implants required, avoiding extended incisions, and ensuring stable three-dimensional alignment between components.

Quantitative evaluation demonstrated a high level of concordance between the planned and postoperative implant positions, with all deviations remaining within clinically acceptable thresholds. Similarly, postoperative orbital volumes closely approximated those of the contralateral healthy side, supporting the effectiveness of the proposed workflow in restoring orbital anatomy. These radiological findings were mirrored by excellent clinical outcomes, with complete resolution of diplopia in all patients and satisfactory correction of enophthalmos in the vast majority of cases.

From a clinical perspective, the proposed two-piece patient-specific implant offers a reproducible solution for managing complex three-wall orbital fractures that are otherwise associated with unpredictable outcomes. The modular design facilitates safe insertion through limited exposure, reduces intraoperative manipulation of orbital contents, and eliminates the need for implant reshaping. This approach may be applicable to secondary reconstructions or complex defects following tumor resection, where precise orbital contour restoration is essential.

Future research should focus on comparative studies evaluating this technique against alternative reconstructive strategies, including preformed meshes and single-piece PSI, as well as multicenter investigations to confirm its generalizability. Further analysis of operative time, cost-effectiveness, and long-term outcomes would also be valuable in defining the role of modular PSI systems in routine orbital trauma practice.

### Limitations

This study has inherent limitations related to its retrospective design and relatively small sample size, which reflects the rarity of three-wall orbital fractures. The absence of a control group prevents direct comparison with alternative reconstructive strategies. Therefore, no claims of superiority can be made based on the current data. The study should therefore be interpreted as a Level IV therapeutic case series and feasibility investigation. Nevertheless, the present study was designed as a feasibility and proof-of-concept investigation aimed at evaluating a novel two-piece patient-specific implant in the challenging clinical scenario of three-wall orbital fractures. The consistency of the radiological accuracy and favorable clinical outcomes observed across this cohort supports the feasibility, safety and reliability of the proposed approach and highlights its potential value in highly complex orbital reconstructions.

## 5. Conclusions

This study presents a novel interlocking two-piece patient-specific implant specifically designed for the reconstruction of complex post-traumatic three-wall orbital fractures. By integrating digital preoperative planning with a modular implant design, this approach allows accurate, reproducible restoration of orbital anatomy through limited surgical exposure.

While further comparative studies are required, this modular two-piece PSI configuration appears to represent a reproducible and technically sound option for the management of selected complex three-wall orbital fractures.

## Figures and Tables

**Table 2:** Orbital Reconstruction Accuracy Assessment and Clinical Outcomes.

Accuracy of Orbital Reconstruction	(Mean ± SD)
Implant deviation	0.982 ± 0.107 (95% CI:0.927–1.037)
Orbital Volume Difference *	1.371 ± 0.176 (95% CI: 1.280–1.461)
**Post-operative Clinical Findings**	***n* (%)**
Diplopia	0 of 17 (0%)
Enophthalmos	2 of 17 (11.8%)
**Major Complications**	0 of 17 (0%)

* Difference between unaffected and reconstructed orbital volume.

## Data Availability

Additional details regarding the data supporting the reported results can be made available upon request to the authors.
